# Supplementary figures and images for: Determining resources and capabilities in complex context: A decision-making model for banks
Source: PLoS One. 2025 May 20;20(5):e0323735. doi: 10.1371/journal.pone.0323735 (PMC12091779; doi:10.1371/journal.pone.0323735)

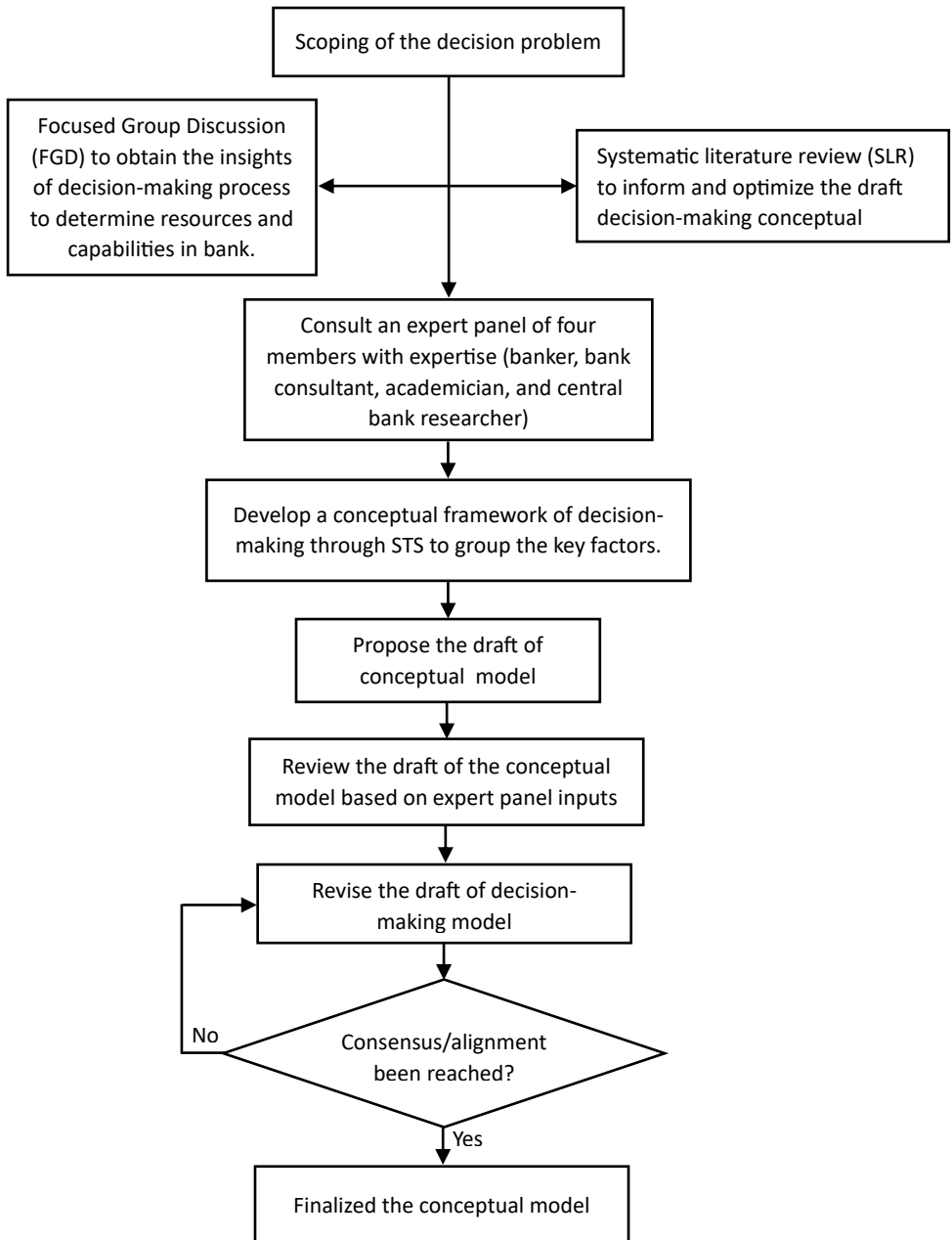

**Fig 2. Research Methodology of Conceptual Model Development.**

Supplement: S2 Fig — (PDF) [file pone.0323735.s002.pdf]

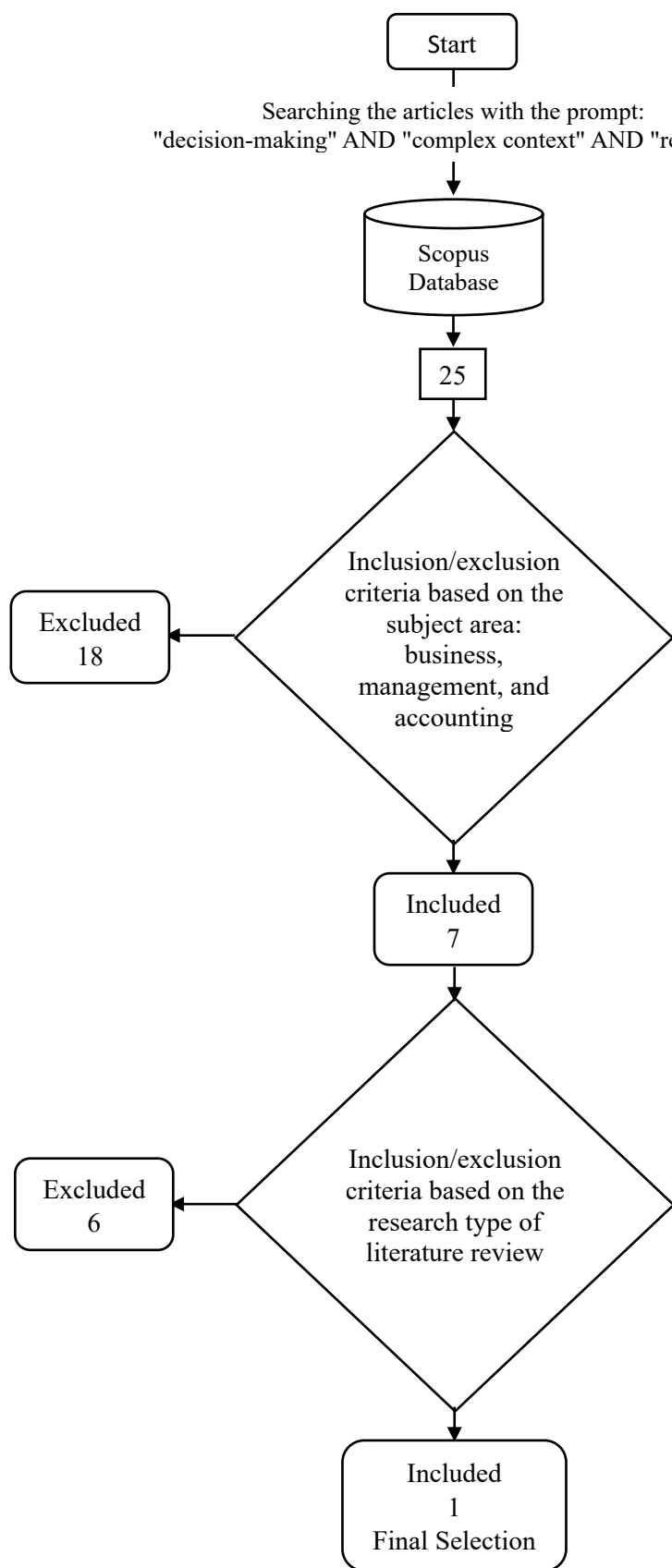

**Fig 3. Inclusion/exclusion searching SLR articles.**

Supplement: S3 Fig — (PDF) [file pone.0323735.s003.pdf]

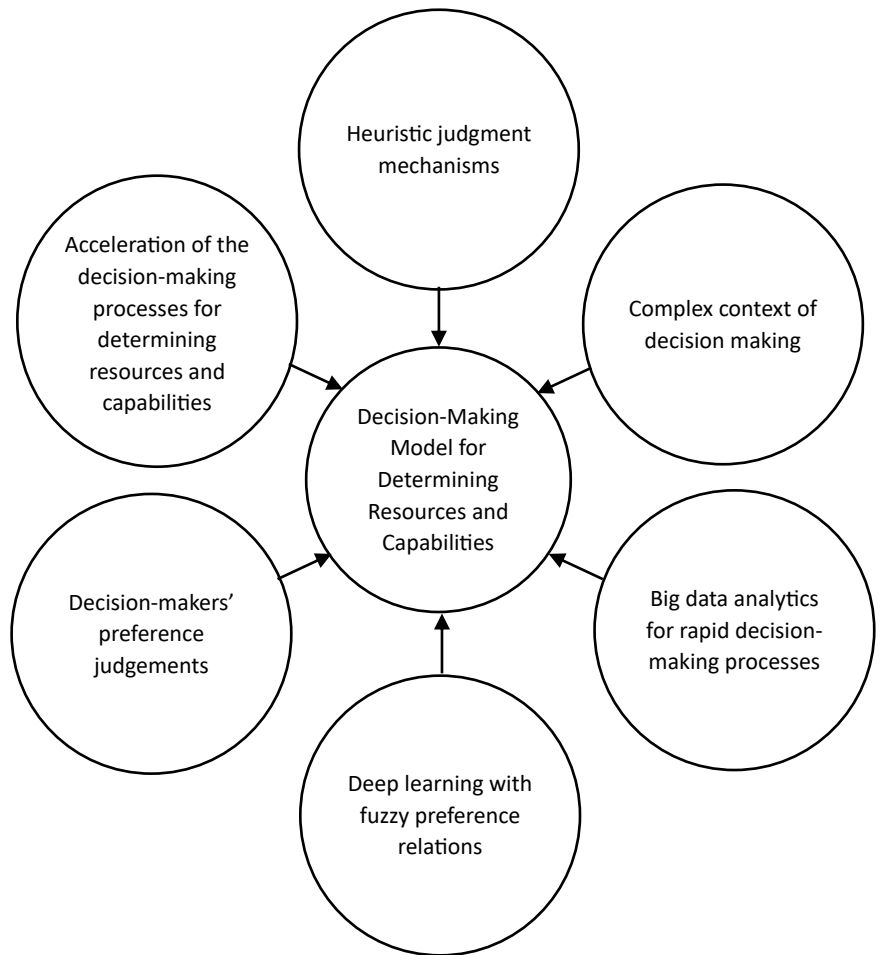

**Fig 5. Proposed conceptual framework of the decision-making model development.**

Supplement: S5 Fig — (PDF) [file pone.0323735.s005.pdf]

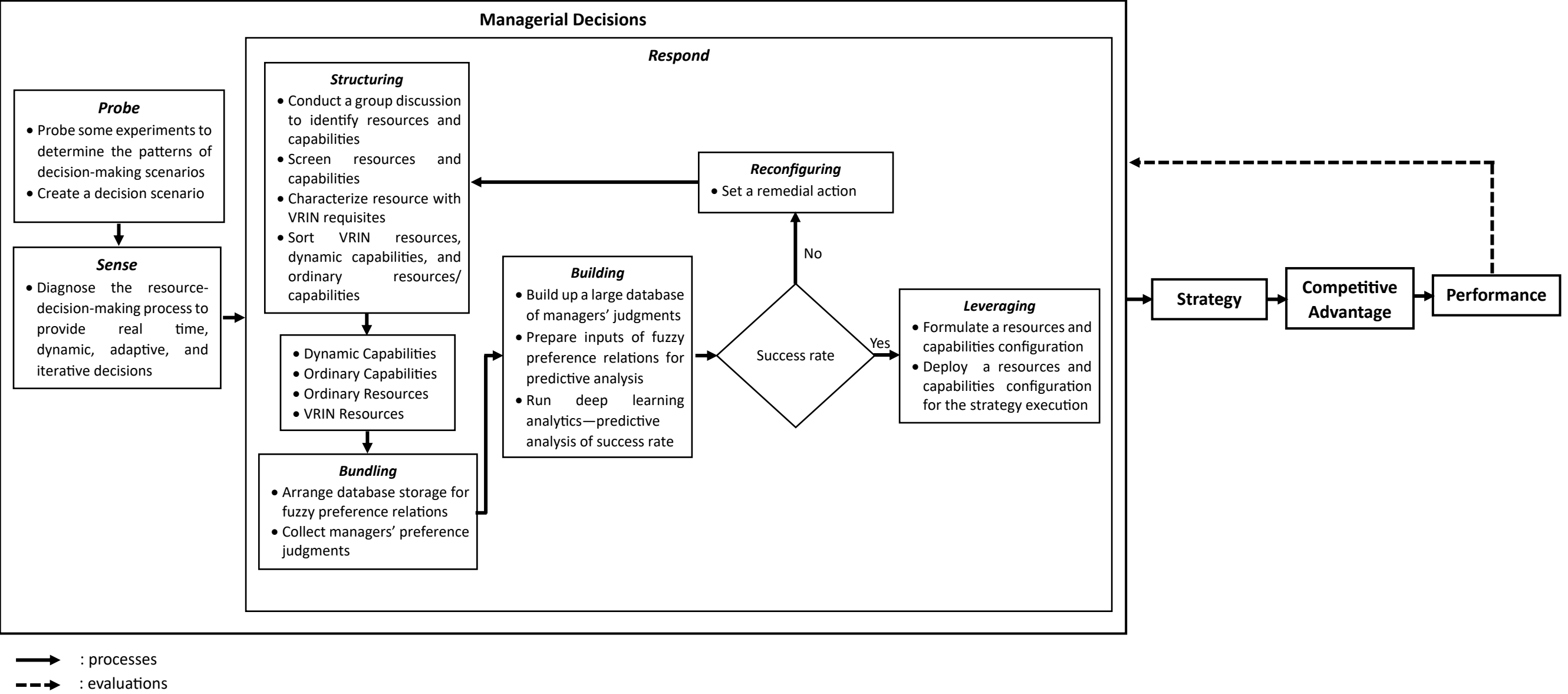

Fig 6. The draft of the decision-making model for determining banks' resources and capabilities

Supplement: S6 Fig — (PDF) [file pone.0323735.s006.pdf]

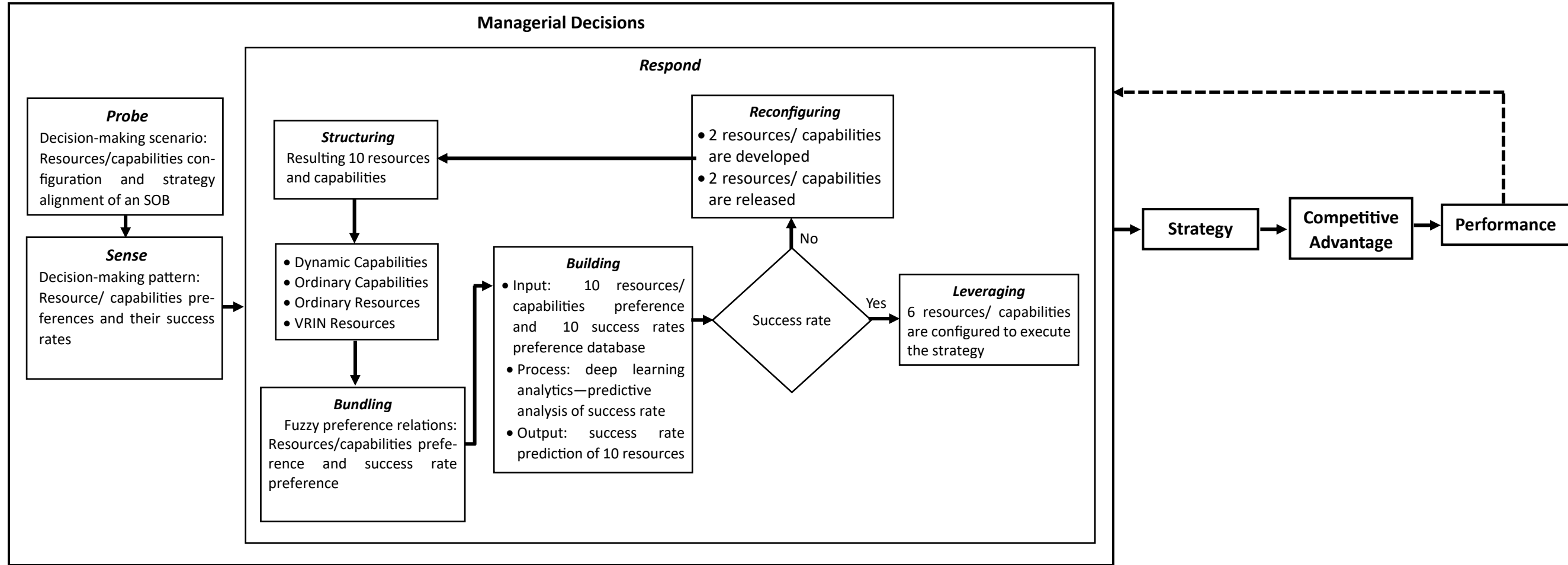

Fig 8. Implementation of the proposed decision-making model in an SOB.

Supplement: S8 Fig — (PDF) [file pone.0323735.s008.pdf]
