# Supplementary material for: Determining resources and capabilities in complex context: A decision-making model for banks
Source: PLoS One. 2025 May 20;20(5):e0323735. doi: 10.1371/journal.pone.0323735 (PMC12091779; doi:10.1371/journal.pone.0323735)
Supplement: S4 Fig — (PDF) [file pone.0323735.s004.pdf]

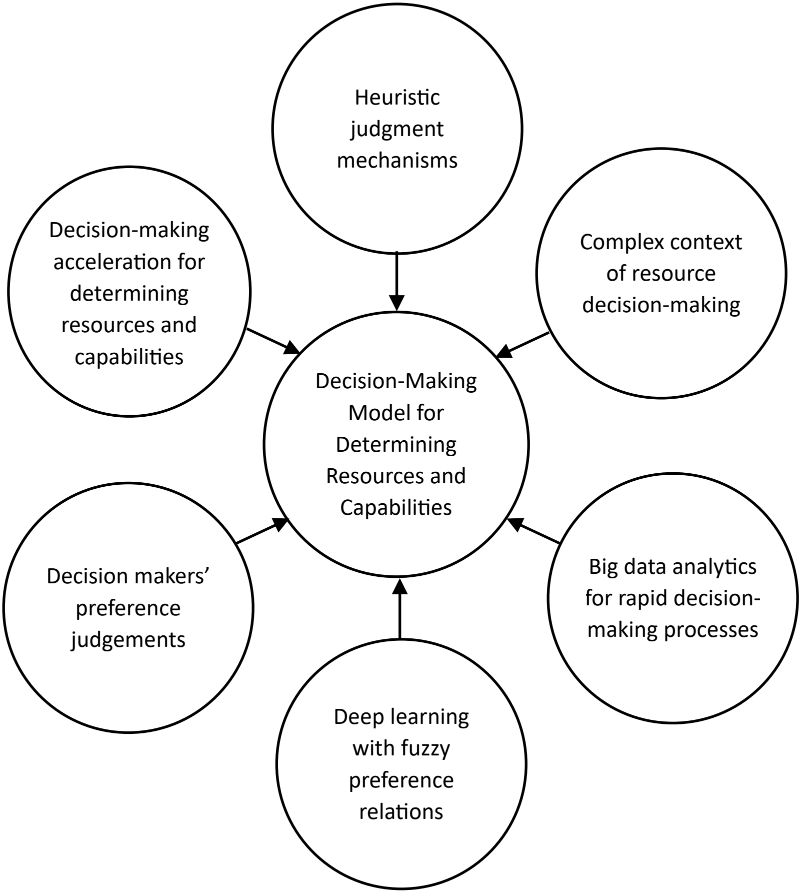

Fig 4. A conceptual framework of the decision-making for determining resources and capabilities.
