# Supplementary material for: Determining resources and capabilities in complex context: A decision-making model for banks
Source: PLoS One. 2025 May 20;20(5):e0323735. doi: 10.1371/journal.pone.0323735 (PMC12091779; doi:10.1371/journal.pone.0323735)
Supplement: Attachment — Submitted filename: PONE-D-24-23190-R1-COMPLETED REVIEW.pdf [file pone.0323735.s011.pdf]

<table>
<tr><th colspan="3">PLOS ONE REVIEW ASSIGNMENT</th></tr>
<tr><td>Manuscript</td><td>:</td><td>PONE-D-24-23190-R1</td></tr>
<tr><td>Prologue</td><td></td><td>Thank you for the opportunity to review this paper. This topic is important from the perspective of how decision-making should be done or approached in a rapid change of the environment based on the Cynefin Framework.</td></tr>
<tr><td>Abstract</td><td>:</td><td>The abstract seems to be the same as the previous draft, where it lacks an explanation of the potential problems when it discusses the gaps in dynamic capabilities. It makes the background of the research discussed in the abstract was not clear.</td></tr>
<tr><td>Introduction</td><td>:</td><td>1. The revised version has not addressed the concerns raised in the previous review, such as:<br>• Lines 63-65 - The authors should explain briefly the rationales for the previous articles' conclusions and how those previous findings support and are relevant to the proposed study.<br>• Lines 65-67 – same inputs as above. There is no explanation or brief discussion related to previous articles in the proposed study. It will strengthen the discussion in the Introduction if the phenomenon and/or background are clearly highlighted rather than cited, as well as the importance of the constructs contemplated in the research model (i.e., if all these relationships contemplated in the study have been confirmed in previous studies, then the novelty of the study could be questionable, aside from the different context where this proposed study is conducted).<br>• Lines 68-69 - It was suggested that organizations use the Cynefin Framework to understand the environment, but there was no brief discussion of the rationales for such a recommendation. Further, the authors should clarify that such a framework was not used in the banking industry so that this proposed study would confirm that such a framework fits the context where this study was conducted.<br>• Lines 68-72 – It consisted of redundant sentences.<br>• Lines 79-87 - The discussions in this section indicate the insufficiency of the dynamic capabilities framework compared to the Cynefin framework. They lack clear supporting arguments based on the previous articles and the context of the frameworks. The authors should provide strong arguments that show evidence that the dynamic capabilities framework did fail during the COVID-19 pandemic. The absence of this solid argument on the insufficiency of dynamic capabilities to the other framework would make this section weak. The authors did not address specifically and clearly what kind of disputes are stated in this section.<br>• Lines 88-99—The examples presented in this section need to be highlighted as relevant and evidence that the dynamic capabilities framework</td></tr>
</table>

| Manuscript | : | PONE-D-24-23190-R1 |
|---|---|---|
| | | failed. On the contrary, the examples provide arguments that the conditions were given and there was no room to maneuver from the decision-making aspects of the company's top management. These are not good examples to highlight the issues of the dynamic capabilities framework vs. the proposed framework from a decision-making perspective due to the given nature of the environment where firms must adhere to such regulations.<br>2. The structure of the introduction needs to be revisited, particularly its contents, which can be significantly improved. The introduction section should focus on the background and/or phenomenon underlying the reasons for conducting this study. In the current stage of the paper, the Introduction section was not written in a structured and systematic discussion. The authors did not support their arguments in indicating that the decision-making framework based on the dynamic capabilities framework failed to respond effectively during the rapid change of the environment, such as the COVID-19 pandemic. Further, the Cynefin framework was just used without clear discussions on the rationales for using such a framework. Further, the research gaps need to be addressed more clearly. Decision-making in an organization is not only affected by the organizational capabilities (such as the use of dynamic capabilities framework) but also by the organizational design where such a firm is organized. The authors seem to ignore the importance of organizational design in the decision-making process in an organization, irrespective of its capabilities. The objective of the study needs to be expanded in the Introduction section rather than outlining the findings.<br>3. The authors' discussion of complex vs. complicated situations was confusing if they discussed the environment in the context of strategic management. Suppose those situations or environments are related to the Cynfine Framework. In that case, the four situations need to be discussed briefly, and the rationales for focusing on only two warrant an explanation. How the authors differentiate the commonly discussed “environment” in the context of strategic management, i.e., munificence, dynamism, and complexity, with their “environment” under the Cynfine Framework also warrants an explanation. Otherwise, it isn't very clear from the perspective of strategic management or from the behavioural strategy’s perspective.<br>4. Some of the inferences or claims in the Introduction section (e.g., lines 80-90) should be supported by theoretical discussions.<br>5. The research objective could be clarified as to whether it would like to enhance the Cynfine Framework or create a brand-new model for decision-making during a rapid |

| PLOS ONE REVIEW ASSIGNMENT | | |
|---|---|---|
| Manuscript | : | PONE-D-24-23190-R1 |
| | | change in the environment. It is not very vague at the moment.<br><br>Please revert to the first review provided prior to this R1 version. The introduction section has not been improved to make a convincing argument that the proposed study is worth exploring. The paper needs to have major revisions not only to rewrite or paraphrase the existing paper but also the contents of the paper need to be revisited and revised accordingly to make this paper more interesting and scholarly stronger. |
| Literature Review | : | Please revert to the inputs in the previous review, which do not seem to have been incorporated into the current revised version (R1). Below are the same inputs provided in the last review.<br><br>1. The literature review needs to be more concise and relevant to the main topic of the research. For example, the authors took a winded discussion on the RBT and Teece's dynamic capabilities before they tried to relate to the decision-making process in the rapid change of the environment.<br>2. The Cynfine Framework warrants further elaboration to ensure that the "situation" referred to in this research is clearly addressed and explained with their relevant theoretical discussions.<br>3. The authors need to integrate the RBT-Dynamic Capabilities Framework, Decision-Making Mechanism & Socio-technical System Theory, Bounded Reality, and Fuzzy Preference Relations in the Heuristic Decision-Making Model into a conceptual framework. Otherwise, the literature review looks unfinished, and there is no objective of having those discussions without integration into one conceptual model framework.<br>4. The discussions of mismatched context, inappropriate treatment, and strategic alignment issues need to be revisited to address a thorough analysis as to what basis the authors came to such inferences that these are the three issues faced by the decision-making employed the dynamic capabilities. Because, intuitively, most of the decision-making related to the changing of the environment would have those issues irrespective of the approaches or frameworks used to explain such decision-making.<br><br>The paper needs major revisions, not only to rewrite or paraphrase the existing paper but also to revisit and revise its contents accordingly to make it more interesting and scholarly. |
| Materials & Methods | : | Please revert to the inputs provided in the previous review, which do not seem to have been embedded in the current revised version (R1). Below are the same inputs provided in the last review.<br><br>1. The authors need to briefly explain the methodology used in their research and not just put the citation with no further |

| PLOS ONE REVIEW ASSIGNMENT | | |
|---|---|---|
| Manuscript | : | PONE-D-24-23190-R1 |
| | | explanation. In fact, the authors used these methods throughout the paper, and the concept discussed was challenging to understand. The discussions in this section lack details and logical explanations as to the rationales for using such a particular method.<br>2. The authors should have explained in more detail each of the actions taken in operationalizing the research methods, i.e., scoping, SLR, and FGD. Further, the authors stated that they conducted in-depth interviews during the FGDs but did not explain how they did the triangulation on such in-depth interviews and what the results were. There are many cases in which the authors seem to assume that the readers would have understood the details of what they have in their minds.<br><br>The paper needs major revisions, not only to rewrite or paraphrase the existing paper but also to revisit and revise its contents accordingly to make it more interesting and scholarly. |
| Results & Discussions | : | The analysis of the method's results applied in this research was not rigorous enough to provide solid and robust support for the research's objective, which is to create a new decision model in the context of rapid environmental change. For example, the authors indicated that they did SLR, but there was no in-depth analysis or discussion of the results of this SLR and how they relate to their proposed model.<br><br>The paper needs to have major revisions not only to rewrite or paraphrase the existing paper but also the contents of the paper need to be revisited and revised accordingly to make this paper more interesting and scholarly stronger. |
| Conclusion | : | The conclusion of the study is weak, and this research is based on only one bank to make it a general finding, particularly in creating a new decision-making model. A more rigorous literature review and research methodology warrant making this research more palatable for scholarly articles in the area of strategic management. The authors entirely ignore organizational design and culture when proposing a new decision-making model in response to the rapidly changing environment. The context where the research was conducted also has its unique characteristic, which is that it is a state-owned enterprise. Therefore, the phenomenon presented may not be only due to the inappropriate use of the decision-making model employed by the dynamic capabilities framework. Still, it could be the organizational characteristics of the context where the authors conduct their research. |
| Epilogue | : | The authors need to revisit their phenomenon, literature review, and the strength of its methods in their proposed research. With its current format and structure, this paper is far from being |

| PLOS ONE REVIEW ASSIGNMENT | | |
|---|---|---|
| Manuscript | : | PONE-D-24-23190-R1 |
| | | acceptable for scholarly publication. Good luck with the research! |
| **Overall Review Results** | **:** | **Reject** |
