# Supplementary material for: Determining resources and capabilities in complex context: A decision-making model for banks
Source: PLoS One. 2025 May 20;20(5):e0323735. doi: 10.1371/journal.pone.0323735 (PMC12091779; doi:10.1371/journal.pone.0323735)
Supplement: Attachment — Submitted filename: Comments and responses Plos One revision.docx [file pone.0323735.s012.docx]

**Comments and responses:**

**Editor**

| **No.** | **Comments** | **Responses** | **Line location** |
| --- | --- | --- | --- |
| 1. | The paper ***is nearest to being rejected***; however, as I see ***some good contributions***, the paper needs to be revised based on comments. | Thank you very much. |  |
| 2. | The following must be done: ***define the study framework in detail and specify the inclusion and exclusion of literature in detail.*** | We revised forepart of introduction section and add the relate works section to highlight the Cynefin framework as the study framework | Line 59 – 87  Line 207 - 229 |
| 3. | The ***abstract*** must be rewritten to ***include the methodology. Questions*** should be ***clearly stated*** in the ***introduction*** and then answered in ***detail in the SLR.*** | 1. We rewrote the abstract section completely with methodology. 2. We added 5 paragraphs to explain the research questions 3. We already detailed the SLR to add the searching process of SLR article, table of detailed SLR article, and research agenda | Line 33 – 55  Line 143 – 185  Line 456 – 487 |
| 4. | Through a comprehensive review, you must design ***a table that shows the proposals for future research*** in terms of the ***descriptive*** and ***thematic aspects***. | We added Table 1. SLR article and research agenda and Fig 3. Inclusion/exclusion searching SLR articles | Line 473 - 483 |
| 5. | You must provide ***a summary of the literature***, stating the ***most important determinants*** in detail based on the ***literature***. | We added the explanation selected SLR article and the findings | Line 475 - 487 |
| 6. | ***Re-extract*** the figures explicitly. | We processed to re-extract the figures and attached them |  |
| 7. | Additional response by author | We removed 1 author due to campus regulation reasons | Line 7 – 23 |

**Reviewer #1**

| **No.** | **Comments** | **Responses** | **Line location** |
| --- | --- | --- | --- |
| 1. | Is the manuscript technically sound, and do the data support the conclusions?  *Answer*: **No** | We rewrote the methodology structure to strengthen the finding of a proposed decision-making model. | Line 324 - 820 |
| 2. | Has the statistical analysis been performed appropriately and rigorously?  *Answer*: **N/A** | Yes, it is. This research is a qualitative study. | Line 325 |
| 3. | Have the authors made all data underlying the findings in their manuscript fully available?  *Answer*: **Yes** | Thank you |  |
| 4. | Is the manuscript presented in an intelligible fashion and written in standard English?  *Answer*: **No** | We enhanced the grammar and improve the academic writing |  |
| 5. | Review Comments to the Author  It is ***an interesting topic*** on the decision-making model in the rapid change of the environment. However, the ***development of the background***, ***literature review***, ***methods*** and the ***results*** are ***weak*** and need to be revisited at its entirety. | 1. We repaired the introduction with additional explanation with additional explanation regarding research background and research questions 2. We strengthened the relates works section with the Cynefin framework 3. We restructured the methodology | Line 59 – 87 and Line 143 – 185  Line 207 – 229  Line 324 - 820 |

**Reviewer #2**

| **No.** | **Comments** | **Responses** | **Line location** |
| --- | --- | --- | --- |
| 1. | Is the manuscript technically sound, and do the data support the conclusions?  *Reviewer 2*: **Partly** | We rewrote the methodology structure to strengthen the finding of a proposed decision-making model. | Line 324 - 820 |
| 2. | Has the statistical analysis been performed appropriately and rigorously?  *Answer*: **Yes** | This research is a qualitative study. | Line 325 |
| 3. | Have the authors made all data underlying the findings in their manuscript fully available?  *Answer*: ***Yes*** | Thank you |  |
| 4. | Is the manuscript presented in an intelligible fashion and written in standard English?  *Answer*: **Yes** | Thank you |  |
| 5. | Review Comments to the Author   1. For the proposed decision-making model, the authors should provide more details on the validation process, including sensitivity analysis or testing the model's applicability in different contexts. 2. The paper also mentions the use of fuzzy preference relationship assessment and big data analysis. The authors should describe the specific steps of these analytical methods, including data preprocessing, model training, and interpretation of results. | 1. We added the explanation for validation model   We detailed this model implementation in a case study of an Indonesian state-owned bank.   2. We detailed fuzzy preference relation and big data analysis in the explanation of the table of the decision-making development complies a proposed conceptual framework and its explanation | Line 786 – 800  Line 822 – 871  Line 708 – 752 |
