# Supplementary material for: Determining resources and capabilities in complex context: A decision-making model for banks
Source: PLoS One. 2025 May 20;20(5):e0323735. doi: 10.1371/journal.pone.0323735 (PMC12091779; doi:10.1371/journal.pone.0323735)
Supplement: Attachment — Submitted filename: Comments and responses Plos One revision2.docx [file pone.0323735.s013.docx]

**Comments and responses:**

**Reviewer #1**

| **No.** | **Comments** | **Responses** | **Line location** |
| --- | --- | --- | --- |
| 1. | If the authors have adequately addressed your comments raised in a previous round of review and you feel that this manuscript is now acceptable for publication, you  may indicate that here to bypass the “Comments to the Author” section, enter your conflict of interest statement in the “Confidential to Editor” section, and submit your  "Accept" recommendation.  Answer: **No Response** | We have revised the manuscript based on reviewer comments. | All lines |
| 2. | Is the manuscript technically sound, and do the data support the conclusions?  *Answer*: **No** | We have revised the SLR article table and FGD results outline table to suit the needs of developing a decision-making model and provide additional information for continuity between data and conclusions. | Line 490 - 535 |
| 3. | Has the statistical analysis been performed appropriately and rigorously?  *Answer*: **Yes** | Thank you |  |
| 4. | Have the authors made all data underlying the findings in their manuscript fully available?  *Answer*: **Yes** | Thank you |  |
| 5. | Is the manuscript presented in an intelligible fashion and written in standard English?  *Answer*: **No** | We have rewritten the manuscript to revise the English grammar and give it an academic tone. | All lines |
| 6. | Review Comments to the Author  Most of the inputs in the previous review have not been addressed. The revisions were done by paraphrasing the previous paper, but not the substance of the paper as highlighted in the review. If the authors have different of opinions, then they should be highlighted in the rebuttal notes to the reviewers. | We have replaced the contents of Table 1 with substantive material to develop the decision-making model in the SLR article. | Line 490 - 507 |

**Reviewer #2**

| **No.** | **Comments** | **Responses** | **Line location** |
| --- | --- | --- | --- |
| 1. | If the authors have adequately addressed your comments raised in a previous round of review and you feel that this manuscript is now acceptable for publication, you  may indicate that here to bypass the “Comments to the Author” section, enter your conflict of interest statement in the “Confidential to Editor” section, and submit your  "Accept" recommendation.  Answer: **All comments have been addressed** | Thank you |  |
| 2. | Is the manuscript technically sound, and do the data support the conclusions?  *Answer*: **Yes** | Thank you |  |
| 3. | Has the statistical analysis been performed appropriately and rigorously?  *Answer*: **Yes** | Thank you |  |
| 4. | Have the authors made all data underlying the findings in their manuscript fully available?  *Answer*: **Yes** | Thank you |  |
| 5. | Is the manuscript presented in an intelligible fashion and written in standard English?  *Answer*: **Yes** | Thank you |  |
| 6. | All questions have been modified specifically to enhance clarity and relevance to the study. The revisions reflect a thoughtful consideration of the feedback provided during the initial review process. | Thank you |  |

**Reviewer #3**

| **No.** | **Comments** | **Responses** | **Line location** |
| --- | --- | --- | --- |
| 1. | If the authors have adequately addressed your comments raised in a previous round of review and you feel that this manuscript is now acceptable for publication, you  may indicate that here to bypass the “Comments to the Author” section, enter your conflict of interest statement in the “Confidential to Editor” section, and submit your  "Accept" recommendation.  Answer: **No Response** | We have taken note of the comments and made improvements based on them. | Line 337 - 972 |
| 2. | Is the manuscript technically sound, and do the data support the conclusions?  *Answer*: **Partly** | We have re-detailed the parts that are less clear and connected. | Line 324 - 820 |
| 3. | Has the statistical analysis been performed appropriately and rigorously?  *Answer*: **No** | Statistical analysis has been demonstrated in the technical review to implement the design of the decision-making model. | Line 894 - 902 |
| 4. | Have the authors made all data underlying the findings in their manuscript fully available?  *Answer*: **Yes** | Thank you |  |
| 5. | Is the manuscript presented in an intelligible fashion and written in standard English?  *Answer*: **Yes** | Thank you |  |
| 6. | Mochammad Ridwan Ristyawan and colleagues present a conceptual model aimed at assisting bank managers in decision-making under conditions of volatility, uncertainty, complexity, and ambiguity, an issue of significant practical relevance. The integration of dynamic capabilities, socio-technical systems (STS), and big data analytics (BDA) provides a novel theoretical foundation. However, while the research framework is ambitious and methodologically rigorous in certain aspects, several critical issues must be addressed to enhance the theoretical contribution, methodological transparency, and practical applicability.  **A) Major Concerns:**  **1. Methodological Rigor:**  One key concern is the methodological rigor of the model development.  In the Functional Requirement Definition (FRD) section (Table 2 in the results), the interview outlines focus heavily on the importance of addressing the problem but provide insufficient detail on the conceptual model itself. This discussion could be better positioned in the discussion section rather than in the methodological framework.  **2. Model Components:**  It is unclear whether all six components of the proposed conceptual framework are essential.  Are all six equally critical in different decision-making contexts, or do they vary in priority depending on the situation?  A more nuanced discussion on the relative importance of these components would strengthen the framework.  **3. Empirical Evidence:**  A significant limitation is the lack of strong empirical support for the model. In the mini-case simulation, the authors primarily describe processes, such as stating that " the BOD analyzes resource and capability requirements for the latest strategy," but do not provide concrete evidence demonstrating the model’s effectiveness.  Is there empirical data showing that applying the conceptual framework leads to measurable improvements in banking operations?    For instance, is there a pre- vs. post-implementation comparison, and if so, what is the effect size?  Furthermore, the mini-case study is narrow in scope and lacks comparative analysis with other banks or industries. A multi-case approach would enhance the model’s generalizability.  **4. Implementation Challenges:**  Another critical issue is the practical implementation of the model.  How can bank managers integrate this framework into real-world decision-making processes?  The authors need to provide more concrete guidance or discussion on implementation strategies.  **B) Minor Concerns:**  Presentation of Table 5: Instead of using a table to conceptualize insights from interconnected dimensions, a network graph might offer better clarity. Specifically, the graph’s nodes could represent the relational dimensions, making the structure more intuitive. | A)   1. We have refined the research methodology adopted from the International Society of Pharmacoeconomics and Outcomes Research—Society of Medical Decision Making (ISPOR-SMDM) which consists of 10 phases to develop a decision-making model.   We have revised the content of Table 2, which outlines the interview results with substantive material to develop a decision-making model.   2.   We have explained the proposed conceptual framework to assists the development of decision-making model.  These six concepts are integrated concepts that form a conceptual framework for building decision-making models in complex contexts.  We have added a discussion to outline the importance of these six concepts.   3.   We have refined the case study of Indonesia SOB by providing empirical examples that can be adopted by other banks.  The proposed conceptual framework is intended to build a decision-making model.  For application in banking operations, this study proposes a decision-making model that has been explained statistical analysis and measurement.  Yes, there is. By using the proposed decision-making model, banks quickly address resource and capability preparation compared to David Teece's dynamic capabilities framework.  The proposed decision-making model is equipped with analytical statistics and measurements that allow banks or other industries to apply it.   4.   In the SOB Indonesia case study, bank managers can follow the flow of implementing the decision-making model. We have explained how bank managers carry out the decision-making process step by step.  B)  We have transformed Table 5 into Figure 4. | Line 337 - 458  Line 509 -535  Line 607 - 654  Line 659 - 674  Line 676 - 712  Line 924 - 972  Line 894 - 902  Line 128 -147 and Line 924 - 972  Line 924 - 972  Line 934 - 972  Line 604 |
